# Improved care and survival in severe malnutrition through eLearning

**DOI:** 10.1136/archdischild-2018-316539

**Published:** 2019-07-30

**Authors:** Sunhea Choi, Ho Ming Yuen, Reginald Annan, Michele Monroy-Valle, Trevor Pickup, Nana Esi Linda Aduku, Andy Pulman, Carmen Elisa Portillo Sermeño, Alan A Jackson, Ann Ashworth

**Affiliations:** 1 Human Development and Health, University of Southampton, Southampton, UK; 2 Faculty of Medicine, University of Southampton, Southampton, UK; 3 Department of Biochemistry and Biotechnology, Kwame Nkrumah University of Science and Technology, Kumasi, Ghana; 4 Facultad de Ciencias de la Salud, Universidad Rafael Landivar, Guatemala City, Guatemala; 5 Department of Nutrition and Diets, Hospital Nacional General “Dr. Juan José Fernandez” Zacamil, San Salvador, El Salvador; 6 National Institutes for Health Research Biomedical Research Centre, University of Southampton, Southampton, UK; 7 Department of Population Health, London School of Hygiene Tropical Medicine, London, UK

**Keywords:** severe acute malnutrition, capacity building, eLearning, WHO ten steps

## Abstract

**Background:**

Scaling up improved management of severe acute malnutrition (SAM) has been identified as the nutrition intervention with the greatest potential to reduce child mortality but it requires improved operational capacity.

**Objective:**

To investigate whether an eLearning course, which can be used at scale in resource-poor countries, leads to improved diagnosis, clinical management and survival of children with SAM.

**Design:**

A 2-year preintervention and postintervention study between January 2015 and February 2017.

**Setting:**

Eleven healthcare facilities: nine in Ghana, one in Guatemala, and one in El Salvador.

**Intervention:**

Scenario-based eLearning course ‘Caring for infants and young children with severe malnutrition’.

**Main outcome measures:**

Identification of children with SAM, quality of care, case-fatality rate.

**Methods:**

Medical record reviews of children aged 0–60 months attending eleven hospitals between August 2014 and July 2016, observations in paediatric wards, and interviews with senior hospital personnel.

**Results:**

Postintervention there was a significant improvement in the identification of SAM: more children had the requisite anthropometric data (34.9% (1300/3723) vs 15.9% (629/3953)) and more were correctly diagnosed (58.5% (460/786) vs 47.1% (209/444)). Improvements were observed in almost all aspects of the WHO ‘Ten Steps’ of case-management, and case-fatality fell from 5.8% (26/449) to 1.9% (14/745) (Post-pre difference=−3.9%, 95% CI −6.6 to −1.7, p<0.001).

**Conclusions:**

High quality, interactive eLearning can be an effective intervention in scaling up capacity building of health professionals to manage SAM effectively, leading to a reduction in mortality.

**What is already known on this topic?**

- In countries most affected by malnutrition, training and curricula are outdated or non-existent, leading to mismanagement of malnourished children and many preventable deaths.
- A shortage of specialist trainers constrains the scaling-up of improved diagnosis and treatment.
- eLearning provides opportunities for delivering training at scale.

**What this study adds?**

- Diagnosis and quality of inpatient care were observed to improve with the interactive eLearning course ‘Caring for infants and young children with severe malnutrition’.
- Case-fatality rates fell from 5.8% to 1.9% after training.

## Introduction

Undernutrition is a major cause of death in early childhood and the ability to identify and treat malnourished children effectively is a core competency for paediatricians and related health professionals.^1^ It was a central consideration in setting the Millennium Development Goals and is a critical aspiration for the Sustainable Development Goals. Achieving this at scale, however, remains challenging^2 3^ and to help address this problem the University of Southampton and the International Malnutrition Task Force of the International Union of Nutritional Sciences developed an eLearning course ‘Caring for infants and young children with severe malnutrition’.^4^ This is interactive, scenario-based and designed around a hypothetical nutrition community in which children and their care are the focus for learning. Since being made freely available in 2010, the course has been taken by over 16 000 health professionals, trainees and educators in over 120 countries.

The course has three modules, each taking about 2–3 hours to complete, and is based on WHO guidelines for the management of severe acute malnutrition^5 6^ and is described elsewhere.^7^ The modules include assessing children for chronic and acute malnutrition; pathophysiological changes in malnutrition and consequences for treatment; managing malnourished children using the WHO Ten Steps; inpatient and community-based management; and how to support mothers and carers to prevent recurrence of malnutrition.

In a recently reported 2-year evaluation study, we found that the course improved knowledge, understanding, skills and confidence of health professionals in the assessment, diagnosis and management of children with severe acute malnutrition (SAM).^7^ Many also reported changing their clinical practices. In this paper we examine whether the eLearning course (the intervention) led to actual improvement in the identification and clinical management of severely malnourished children, and to a reduction in case-fatality rates.

## Methods

This preintervention and postintervention study was conducted in Ghana (Ashanti Region), Guatemala, El Salvador and Colombia, and a subset of healthcare facilities that provided paediatric care in these countries was selected for study between January 2015 and February 2017. Preintervention data were collected before the eLearning course was introduced to the health professionals working at these facilities and postintervention data were captured at three time-points: 0–1, 6 and 12 months after the training. See online supplementary file 1 for data collection and study periods.

10.1136/archdischild-2018-316539.supp1 Supplementary data

### Participants and setting

Study participation was voluntary, and healthcare facilities providing paediatric care were eligible to participate. Private facilities were ineligible for study participation, but access to the course was open to all. A mixture of convenience and targeted sampling was used. In Ghana, ten hospitals, urban and rural, in Ashanti region where the country research team was based, were contacted and nine hospitals accepted the invitation and participated in the evaluation. In Latin America, the teaching hospitals affiliated to the researchers’ institutions were targeted and three of four contacted hospitals, one each in Guatemala, El Salvador and Colombia, accepted the invitation. During the study the hospital in Colombia withdrew their permission for review of medical records and observations, and data from this hospital were excluded from the analysis.

In Ghana, the health professionals who participated in the training were mostly doctors/medical officers, nurses and nutritionists. In Latin America, participation was constrained by proficiency in the course language (English) and all the participants were doctors.

After collecting preintervention data, health professionals at these facilities were introduced to the course by the country research team between July and early August 2015. In Ghana letters were sent to the head of each hospital in advance of the training requesting that about 30 staff (especially paediatric staff) be released to attend the introduction. The hospitals contacted their Directorates in the Ghana Health Service informing them of the training opportunity. Those invited for training were internal staff working at the participating hospitals and external staff from other linked health facilities. In Latin America, all paediatric doctors were invited. In each hospital the introduction lasted approximately 1 hour and included how to access the course and its navigation structure. Internet access was good in Latin America and participants were given 3 weeks to complete the course in their own time online. In Ghana few participants had internet access at work or home, so a CD version of the course was installed on laptops brought by the research team and some participants at each hospital, and participants took the course over 2 days.

Apart from the introduction to the course, no other support was provided, and no other concurrent interventions occurred. Access to the course remained available to all staff at the participating facilities, including staff who were unable to participate in the scheduled training.

### Data collection

The study was conducted by three teams of researchers: the UK team led and managed the overall study, and the Ghana and Latin America teams identified the study sites and collected the data. Data collection included abstracting information from medical records, observing clinical practices and interviewing senior hospital personnel. The researchers in Ghana and Latin America had higher education degrees in nutrition. Protocols were developed before the study started to standardise and guide the teams in data collection and management. For medical record data, a Microsoft Excel template with precoded Macros was set up. For observations, a structured form developed for assessing hospitals in Tanzania^8^ was used in which specific components of the WHO Ten Steps for management of SAM are checked against actual practice. Protocols, data collection tools and recording templates were reviewed prior to the study by a specialist in clinical data management and pretested to standardise data collection, entry, cleaning and management. Secure storage for research data and SharePoint group site for project files were set up, and a file management guideline, including naming convention, folder structure and secure upload through Southampton Virtual Environment, was prepared.

### Ability to diagnose SAM correctly

The WHO definition of SAM is weight-for-height <−3SD or mid-upper arm circumference (MUAC) <115 mm (6–60 months) and/or oedema.^9^ Data were abstracted from the actual medical records of (i) all children aged 0–60 months attending six participating hospitals (four in Ghana and two in Latin America) and (ii) children aged 0– 60 months attending malnutrition ward/units in five hospitals in Ghana between August 2014 and July 2016 to see (a) if the requisite data for diagnosing SAM were present and (b) if the diagnosis was correct. The abstracted data were: admission date, date of birth (or age), weight, height (or length), MUAC, bipedal oedema, diagnosis, associated conditions and outcomes. The WHO criteria for identifying SAM were applied to all the collected records of children aged 6–60 months. Children who were reported as cases of SAM were categorised either as ‘matched the WHO definition’ or as ‘false’ (ie, did not match the WHO definition), or as ‘unclassifiable’ due to insufficient data being recorded. Additionally, ‘missed’ cases of SAM were identified. The proportions of matched, false, unclassifiable and missed cases during 12 months preintervention were compared with those for the 12 months postintervention to capture changes in the ability to assess and diagnose SAM correctly.

For children with a diagnosis of acute respiratory infection (ARI) or gastroenteritis, the information was also used to explore whether there was an increase in the proportion whose malnutrition status was checked on admission postintervention, as the usual treatment for these conditions is made more difficult when there is coexisting SAM.

### Quality of clinical care

At each hospital (except El Salvador) the care of SAM patients was observed at three time points (preintervention, 6 and 12 months postintervention). The hospital in El Salvador was observed only postintervention. Observations were unannounced and were for 2–3 hours in Ghana, 8–9 hours in Guatemala and 24 hours in El Salvador. The purpose was to determine any changes in the quality of care as a result of the intervention. Aspects of interest included time from arrival to admission, feeding practices, dispensing of medications, hygiene, play activities and education sessions for carers. Observations were also made of the accuracy of anthropometric measurements and feed preparation, and availability of WHO reference charts and guidelines. Some information that could not be observed, such as time of arrival, was obtained by questioning carers and staff.

### Case-fatality rates

The percentage of children with SAM who died was calculated for the 12-month period before the intervention and for 12 months after. Medical records of children (a) aged<6 m with reported SAM and (b) 6–60 months assessed against WHO criteria for SAM were used for each period and the percentage who died was compared.

### Impact on referral rates

The percentage of SAM admissions referred to a tertiary hospital was calculated for the 12-month period before the intervention and for the 12-month period after.

### Operational and policy changes

Semistructured key informant interviews were conducted at four time points (preintervention and 0–1, 6 and 12 months postintervention) with senior hospital personnel who were responsible for paediatric care at the participating hospitals, including medical superintendent (1), directors of district/municipal health services (2), senior nurses (5), heads of paediatrics (2), district nutrition directors (2) and senior nutrition officers (4). The aim was to capture their approach to SAM management, their perceived impact of the intervention, and any subsequent operational and policy changes. Directors of District Health Services in Ghana were further interviewed to capture overall changes in the management of SAM in their districts.

### Statistical analysis

Χ^2^ or Fisher’s exact test was performed when assessing the changes in assessment and diagnosis of SAM and SAM-related case-fatality preintervention and postintervention. Χ^2^, Fisher’s exact or two-sample t-test was performed when comparing the nutritional status of SAM cases. Statistical significance was set at 5%. IBM SPSS Statistics for Windows, V.25.0 (IBM Corp.) was used. CI Analysis software^10^ was used to calculate the difference in percentages and 95% CIs.

## Results

Table 1 shows the types of participating healthcare facilities. These ranged from district to national hospitals. Most were district level hospitals.

**Table 1:** Types of participating healthcare facilities in the study

Variable	Ghana	Guatemala	El Salvador	Total
Type of healthcare facility* —Total	9	1	1	11
District, government hospital†	3	–	–	3
District, mission hospital‡	3	–	–	3
Municipal, mission hospital‡	1	–	–	1
Regional, government hospital†	1	–	–	1
National hospital	–	–	1	1
Maternal and child hospital§	1	1	–	2
Type of ward—Total	9	1	1	11
Malnutrition	1	–	–	1
Paediatric malnutrition unit	4	–	–	4
Paediatric	3	–	1	4
General (for both adults and children)	1	–	–	1
Emergency nutrition department	–	1	–	1
Type of outpatient department—Total	9	–	–	9
Malnutrition	1	–	–	1
Paediatric	7	–	–	7
General	1	–	–	1

*Limited to the hospitals from which the abstraction of data from medical records was carried out. The eleven participating hospitals are: Maternal and Child Health Hospital (MCHH), St Michael’s Hospital (SMiH), Ejura Government Hospital (EGH), Agogo Presby Hospital (APH), St Patrick’s Hospital (SPH), St Martin’s Hospital (SMaH), Konongo Government Hospital (KGH), Kumasi South Hospital (KSH) and Mankranso Government Hospital (MGH) in Ghana; Hospital Juan Pable II (HJP) in Guatemala; and Hospital Nacional Zacamil (HNZ) in El Salvador.

†Hospitals that are funded by government (Ghana Health Service) and located in Ashanti region offering primary care service: EGH, KGH and MGH (district hospitals) and KSH (regional hospital).

‡Hospitals that are in partnership with Christian Health Association of Ghana (CHAG): SMH, APH, SPH and SMaH.

§Includes MCHH in Ghana. MCHH is a government hospital in Kumasi but it offers a dedicated care for malnourished children and adults. MCHH has SAM only inpatient ward and outpatient department unlike other district/regional government hospitals.

SAM, severe acute malnutrition.

### Health professionals trained

In Ghana, 318 health professionals were trained of whom 141 worked in the participating hospitals. In Latin America 28 doctors were trained. Details of those trained in Ghana are given in online supplementary file 2. Most worked regularly with malnourished children (84.3% (268/318) in Ghana and 96.4% (27/28) in Latin America). Few, however, had received prior training in the management of SAM (29.2% (93/318) in Ghana and 39.3% (11/28) in Latin America), and only 12.3% (39/318) and 21.4% (6/28) respectively had been trained about the WHO Ten Steps.

10.1136/archdischild-2018-316539.supp2 Supplementary data

Postintervention 64.7% (90/139) of the health professionals trained in Ghana reported accessing the course continuously for learning, and 66.2% (92/139) in Ghana and 73.3% (11/15) in Latin America recommended it to their colleagues.

### Ability to assess and diagnose SAM correctly

Table 2 shows that postintervention there was a notable improvement in the proportion of children with the requisite data for assessing SAM (34.9% vs 15.9%, p<0.001) and the number and proportion reported as having SAM almost doubled. There was also an improvement in the proportion of cases correctly diagnosed as having SAM (matched 58.5% vs 47.1%, p<0.001). Many missed cases (both preintervention and postintervention) were from the hospital in El Salvador which considered malnutrition as a complication, rather than a diagnosis (5/24 missed cases preintervention and 44/67 postintervention).

**Table 2:** Changes in the quality of assessment and diagnosis of SAM in eleven participating hospitals for the 12 months preintervention and 12 months postintervention

Category	Preintervention [A]	Postintervention [B]	Difference [B – A] in % (95% CI)	P value
SAM assessment				
Number of cases analysed (0–60 months)*	3953	3723		
<6 months	292	438		
6–60 months	3637	3280		
Number (%) of cases with the requisite measurement data for WHO classification†‡	629 (15.9)	1300 (34.9)	19.0 (17.1 to 20.9)	<0.001
Number (%) of cases reported as SAM§	491 (12.4)	807 (21.7)	9.3 (7.6 to 10.9)	<0.001
<6 months	65 (22.3)	83 (18.9)	−3.3 (−9.4 to 2.6)	0.276
6–60 months	420 (11.5)	719 (21.9)	10.4 (8.6 to 12.1)	<0.001
Number (%) of cases classified based on WHO SAM criteria (6–60 months)	444	786		
Matched SAM	209 (47.1)	460 (58.5)	11.5 (5.6 to 17.2)	<0.001
Unclassifiable SAM	151 (34.0)	135 (17.2)	−16.9 (−22.0 to 11.7)	<0.001
False SAM	60 (13.5)	124 (15.8)	2.3 (−2.0 to 6.2)	0.285
Missed SAM	24 (5.4)	67 (8.5)	3.1 (0.1 to 5.9)	0.045
SAM-related morbidity				
Number (%) of cases diagnosed as acute respiratory infection in whom SAM was identified	44/620 (7.1)	81/742 (10.9)	3.8 (0.7 to 6.8)	0.015
Number (%) of cases diagnosed as gastroenteritis in whom SAM was identified	34/587 (5.8)	95/716 (13.3)	7.5 (4.3 to 10.6)	<0.001

*Includes 24 and 5 cases with no date of birth recorded in preintervention and postintervention respectively.

†The requisite measurement data are: age, gender, MUAC or weight and height, and/or oedema presence.

‡Includes 4 and 3 cases with no date of birth recorded in preintervention and postintervention respectively.

§Includes 6 and 5 reported SAM cases with no date of birth recorded in preintervention and postintervention period respectively.

MUAC, mid-upper arm circumference; SAM,  severe acute malnutrition.

Postintervention there was a significant increase in the proportion of children with ARI in whom SAM was identified (10.9% vs 7.1%, p=0.015) and the same was found for gastroenteritis cases (13.3% vs 5.8%, p<0.001).

### Observed quality of care

Table 3 summarises the observed changes in case-management at ten hospitals (the hospital in El Salvador was excluded as no preintervention observations were made). Improvements were observed in almost all components of the WHO Ten Steps. Time from arrival to admission became shorter, and hygiene improved greatly. Feed preparation, storage and administration of correct type of feed also improved. Preintervention, some hospitals in Ghana used diluted F100 or ready-to-use therapeutic food (RUTF) for stabilisation instead of F75, but postintervention all nine hospitals used F75 and followed the recipe correctly. Hospitals in Latin America used commercial infant formula for stabilisation and rehabilitation rather than F75 and F100, and this continued postintervention. Follow-up after discharge improved.

**Table 3:** Changes in the management of SAM observed in ten participating hospitals* at preintervention and 6–12 months postintervention

Step	Description	Preintervention					6–12 months postintervention					*Difference* (Y)
		Y	I	N	N/O	N/A	Y	I	N	N/O	N/A	
1. Treat/prevent hypoglycaemia	Arrival to admission time (<2 hours)	**4**		5		1	**9**				1	5
2. Treat/prevent hypothermia	Children remain covered	**4**	4	2			**8**	2				4
3. Treat/prevent dehydration	ReSoMal given instead of ORS	**2**		7		1	**4**	6				2
4&6. Correct electrolyte imbalance and micronutrient deficiencies	Salt withheld from additional foods	**3**	1	5	1		**9**	1				6
	CMV or source of potassium, magnesium, zinc and multivitamins given.	**6**		3	1		**9**	1				3
	Iron withheld in the stabilisation phase.	**7**	1	2			**10**					3
	Diuretic not given to treat oedema. (Yes, not given; No, given)	**6**	2	2			**8**			2		2
5. Treat infection	Antibiotics given within 30 min of prescription time	**7**		3			**10**					3
	Antibiotic type and dose given according to prescription	**8**	1	1			**10**					2
5. Hand washing	Staff wash/spray hands between contact with each child	**5**		5			**7**		3			2
	Staff wash/spray hands before preparing feeds	**5**	2	3			**10**					5
	Mothers wash/spray hands before giving feeds	**1**	2	3	2	2	**10**					9
	Soap/spray available for mothers	**4**	1	5			**9**		1			5
5. Ward hygiene	New or sterilised syringes used for each feed given through NG tube	**3**	1	4	1	1	**10**					7
	Cups washed with soap between each feed	**7**	1		2		**10**					3
	Feeding equipment washed with soap between each feed preparation	**5**	1	4			**10**					5
	Clean toilet available for mothers	**4**		6			**10**					6
	Boiled water used to make feeds	**5**		2	1	2	**10**					5
7&8. Feeding	Correct feed type given to each child according to prescription (F75 or F100 or RUTF)	**3**		4	1	2	**9**		1			6
	Correct volume of feed given according to prescription	**5**		4		1	**10**					5
	Feeds given on time (within 15 min of prescription)	**3**	1	4	1	1	**10**					7
	Actual volume taken charted (ie, leftovers charted)	**2**	1	7			**1**		9			-1
	If child vomits, feed reoffered	**4**		4	1	1	**10**					6
	Reluctant feeders encouraged to eat	**4**	2	1	2	1	**10**					6
	Children on F100 fed until full	**6**		1	1	2	**9**				1	2
	Additional foods withheld in stabilisation phase	**5**	3	2			**8**		2			3
9. Sensory stimulation	Colourful pictures/displays on walls	**4**		6			**4**		6			0
	Toys are available in/around beds	**1**		9			**1**		9			0
	Structured play sessions held for children.	**2**		8			**1**		9			-1
10. Follow-up	Mothers given a follow-up letter on discharge†	**4**		6			**4**		6			0
General: Monitoring	Weighing scales present	**6**	1	3			**10**		0			4
	Good technique used to weigh children	**3**	1	5	1		**8**	1	1			5
	Length board present or MUAC tapes available	**4**		6			**9**		1			5
	Good technique used to measure height/length OR to measure MUAC	**3**	1	3	2	1	**8**		1	1		5
General: Ward	Separate ward or ‘corner’ available to treat severe malnutrition	**1**		7		2	**1**		8		1	0
	Charts for each child kept at end of their bed	**3**		7			**8**		2			5
	Equipment on ward in good working order	**6**		3		1	**10**					4
General: Staff	Minimum of one nurse to five children available during day	**4**		6			**10**					6
	Doctor/s visit ward at least once per day outside of ward rounds/emergencies	**7**		1	2		**9**		1			2

*Preintervention observation data were not collected from the participating hospital in El Salvador. The postintervention observation data from this hospital were excluded from the analysis.

†Verbal follow-up instructions were given to mothers at five hospitals at preintervention and six hospitals at 6–12 months postintervention.

CMV, combined mineral and vitamin mix; I, inconclusive/inconsistent; MUAC, mid-upper arm circumference; N, no; NG, nasogastric; N/O, not observed; N/A, not applicable; ORS, oral rehydration solution; ReSoMal, rehydration solution for malnutrition; RUTF, ready-to-use therapeutic food, SAM, severe acute malnutrition; Y, yes.

Postintervention 9/10 hospitals had anthropometric equipment and this functioned properly. Anthropometric techniques gradually improved and WHO reference charts became available. Recipes for F75 and F100, and feed volume charts became accessible.

Aspects of management that showed small or no improvement were having a separate area for SAM cases, using the special rehydration solution for malnutrition, measuring leftover feed and recording actual intake, having separate sinks for washing hands and equipment, and providing play and sensory stimulation. There was a policy in most hospitals to disallow toys for hygiene reasons.

### Impact on case-fatality rate

SAM cases (table 4) were similar preintervention and postintervention in age, anthropometric status and clinical complications.

**Table 4:** Comparability of nutritional status and age of SAM cases (0–60 months)* treated at the eleven participating hospitals during 12 months preintervention and 12 months postintervention

Category	Preintervention [A]	Postintervention [B]	Difference [B – A] in mean or % (95% CI)	P value
Weight-for-height z score			0.1 (−0.2 to 0.4)	0.492†
n	98	261		
Mean (SD)	−4.2 (1.3)	−4.0 (1.3)		
Mid upper arm circumference (MUAC) in mm			−0.1 (−1.8 to 1.6)	0.943†
n	127	259		
Mean (SD)	104.8 (6.8)	104.7 (8.5)		
Oedema presence			−7.1% (−12.1 to 2.6)	0.002‡
n	48/298	55/610		
(%)	(16.1)	(9.0)		
Complications			1.4% (−4.8 to 7.2)	0.654‡
n	75/298	164/610		
(%)	(25.2)	(26.6)		
Age in months			1.0 (−0.3 to 2.2)	0.133†
n	298	610		
Mean (SD)	12.4 (8.5)	13.4 (9.2)		

*SAM cases 6–60 months are those that met WHO criteria (matched and missed cases). For infants aged <6 months, the WHO criteria are less specific but 31 in this age group preintervention and 48 postintervention had anthropometric and/or oedema data and their data are included.

†Two-sample t-test was performed.

‡Χ^2^ test was performed.

SAM, severe acute malnutrition.

**Figure 1:**
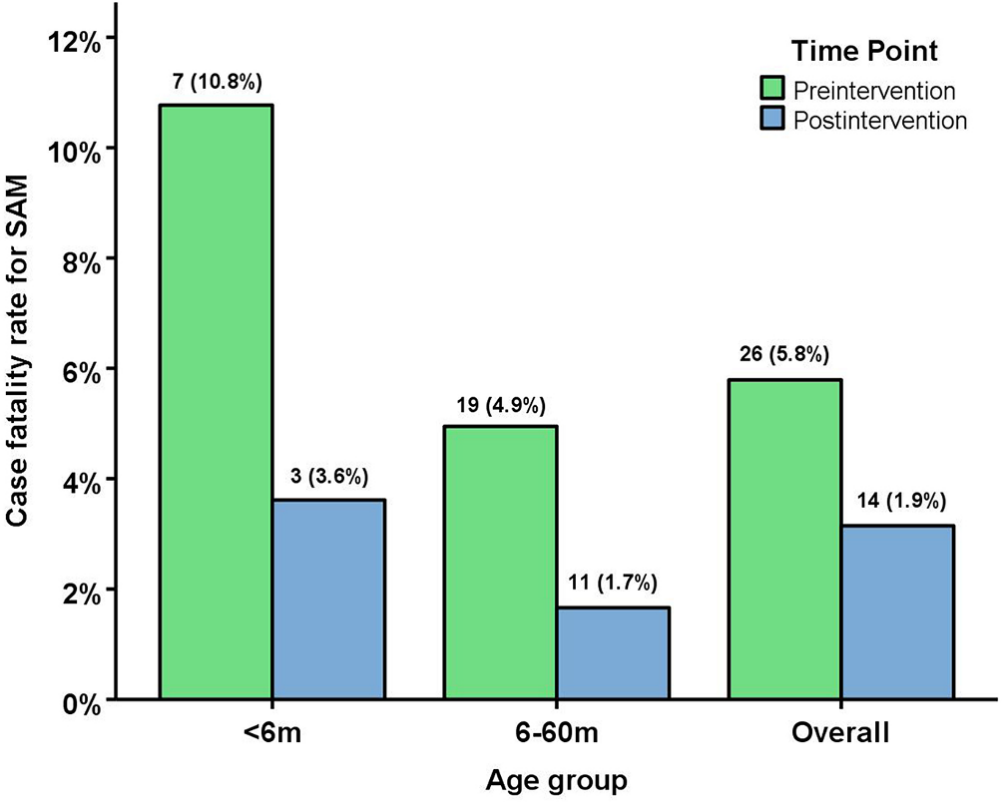
Case-fatality rates for SAM for the 12 months preintervention and 12 months postintervention (<6 months, 6–60 months and Overall). Difference in % between postintervention and preintervention (95% CI) and p value: <6 months (−7.2 (−17.3 to 1.3) *Fisher’s exact* test p=0.106); 6–60 months (−3.3 (− 6.0 to –1.1), Χ^2^  test p=0.002); Overall (−3.9 (−6.6 to –1.7), Χ^2^test p<0.001]. SAM, severe acute malnutrition.

Figure 1 and online supplementary file 3 show that the overall case-fatality rate decreased significantly from 5.8% (26/449) preintervention to 1.9% (14/745) postintervention. Subgroup analysis by type of facility in Ghana (table 5) shows preintervention case-fatality rates ranging from 4.0% (Maternal and Child Health Hospital) to 19.0% (District/regional government hospitals), and the highest case-fatality rate at an individual hospital was 28.6% (4/14). Postintervention, all three types of facility had rates <4%. Online supplementary file 4 shows data for each hospital.

10.1136/archdischild-2018-316539.supp3 Supplementary data

10.1136/archdischild-2018-316539.supp4 Supplementary data

**Table 5:** Subgroup analysis of the numbers (%) of SAM cases (0–60 months) and case-fatality (%) by type of facility in Ghana

Types of facility	n	SAM cases		Case-fatality rate			
		Preintervention	Postintervention	Preintervention [A]	Postintervention [B]	Difference [B – A] in % (95% CI)	P value
District/regional government hospitals*	4	42 (9.7%)	44 (6.5%)	8/42 (19.0%)	0/44 (0.0%)	−19.0 (−33.3 to 6.9)	0.002†
Mission hospitals‡	4	95 (21.8%)	322 (47.5%)	6/95 (6.3%)	12/322 (3.7%)	−2.6 (−9.6 to 1.7)	0.262†
Maternal and Child Health Hospital§	1	298 (68.5%)	312 (46.0%)	12/298 (4.0%)	2/312 (0.6%)	−3.4 (−6.3 to 1.0)	0.005¶
Overall	9	435 (100%)	678 (100%)	26/435 (6.0%)	14/678 (2.1%)	−3.9 (−6.7 to 1.6)	0.001¶

*Hospitals funded by Ghana Health Service. Two hospitals, EGH and KSH, treated SAM preintervention. KGH initiated the treatment of SAM postintervention. MGH did not manage children with SAM preintervention and postintervention.

†Fisher’s exact test was performed.

‡Hospitals that are in partnership with Christian Health Association of Ghana (CHAG): SMiH, APH and SPH managed SAM preintervention. SMaH initiated the treatment of SAM postintervention.

§MCHH in Kumasi offers a dedicated care for malnourished children and adults. MCHH has SAM only inpatient ward and outpatient department. Other district/regional government hospitals in this study manage SAM cases as part of paediatric or general care, and therefore, we analysed the data from MCHH separately from other government hospitals.

¶Χ^2^ test was performed.

APH, Agogo Presby Hospital; EGH, Ejura Government Hospital; KGH, Konongo Government Hospital; KSH, Kumasi South Hospital; MCHH, Maternal and Child Health Hospital; MGH, Mankranso Government Hospital; SAM, severe acute malnutrition; SMiH, St Michael’s Hospital; SMaH, St Martin’s Hospital; SPH, St Patrick’s Hospital.

The percentage of oedematous cases was lower postintervention (table 4). Of these oedematous cases, 16.7% (8/48) died preintervention and 5.5% (3/55) died postintervention (post-pre difference=−11.2%, 95% CI (−24.6 to 1.1), Χ^2^ test p=0.066).

### Impact on referral rates

Preintervention in Ghana, 6.7% (29/435) of children admitted with SAM were referred onwards to a tertiary hospital. Postintervention this fell to 1.3% (9/678); post-pre difference=−5.4, 95% CI −8.2 to −3.0, p<0.001. This reduction coincided with the establishment of malnutrition units at two hospitals and greater confidence of staff to manage SAM competently.

These figures apply only to children admitted to participating hospitals. The largest tertiary hospital in the Ashanti Region is Komfo Anokye Teaching Hospital which was not part of this study. Seriously ill children with SAM, malaria or other morbidities are usually referred to this hospital direct from emergency or outpatient departments and so are never admitted as in-patients at most district hospitals.

### Operational and policy changes

Information from interviews with senior hospital personnel matched the observed changes. The observed changes also matched the reported changes described in the companion paper,^7^ including increased screening for SAM among under-fives and improved identification and treatment of SAM. Greater staff confidence in diagnosis and case-management was also noted. In Ghana, nurses started to prepare F75 and F100 which avoided children missing feeds. Many staff who took the course were reported to have shared their knowledge with colleagues, for example through workshops and using the course for case review in team meetings. One hospital made the course mandatory for all incoming paediatric staff and facilitated copies of the course for personal use. Two hospitals, one mission and one government, established malnutrition units enabling them to treat SAM cases rather than referring them to the tertiary hospital, and one of these was reported to have used the course online for training staff in the paediatric ward and malnutrition unit.

Other reported changes were the introduction of (a) specific discharge criteria, allowing children to stay for longer, and (b) follow-up of discharged children. At community level, procedures were introduced to check if the parent took a referred child to hospital. Excerpts from the interview with a Director of District Health Services are in online supplementary file 5, illustrating the changes reported above.

10.1136/archdischild-2018-316539.supp5 Supplementary data

## Discussion

Three findings in this evaluation are of particular note. First, caring practices were observed to improve. Second, the case fatality rate fell from 5.8% preintervention to 1.9%, even though participating hospitals managed more SAM children themselves postintervention instead of transferring them to tertiary hospitals for treatment. In-service face-to-face training has been shown to benefit quality of care and case-fatality in severely malnourished children^11–13^ but as far as we are aware this is the first time that an eLearning course has been evaluated and found to be effective in improving the identification, clinical management, and survival of children with SAM. This is important because it demonstrates that the eLearning course can scale up the training of health professionals in the management of SAM, which is identified as the key to substantially decreasing case-fatality rates worldwide.^14^ Third, postintervention, twice as many children attending participating health facilities were assessed for SAM. This is important as children with SAM need to be managed differently from other sick children because of their profound metabolic and physiological changes.

Could the improvement in case-fatality rate be due to under-reporting preintervention or over-diagnosis postintervention? These are potential problems if hospital diagnoses are used but were avoided in this study since cases were designated as SAM by the study team using strict WHO criteria on the anthropometric measurement data abstracted from medical records. Consequently, ‘missed’ cases of SAM were included both preintervention and postintervention when calculating case-fatality rates, and ‘incorrectly diagnosed’ cases were excluded. Taking ‘missed’ cases into account also helps avoid potential bias that may arise from the increased case-finding seen in hospital records postintervention.

Confidence in the finding of improved survival is further strengthened by (a) the total number of ‘under-five’ hospital admissions being similar preintervention and postintervention (3953 vs 3723) and (b) the severity of wasting being similar in both time periods (Z-score −4.2 pre vs −4.1 post, p=0.507). The ‘Hawthorne effect’  of changed behaviour as a result of being observed is an unlikely contributor to improvement in survival in this study as comparable observations took place both preintervention and postintervention. Also, relatively little time (maximum 3 days) was spent observing ward practices whereas case fatality rates were calculated over periods of 12 months.

In 2008, the Ghana Ministry of Health adopted the community-based management of acute malnutrition.^15 16^ While the pilot and early phase of implementation were successful in reducing case fatality in communities to 2%, it proved difficult to provide the training and support for capacity building and programme implementation, especially in the phase 2 regions of which Ashanti is part, and identification and management of SAM remained ineffective.^16^ Sensitisation regarding the need for capacity building had thus occurred before the intervention and there was cooperation and encouragement from hospital managers and directors of district Ghana Health Service towards the eLearning course. Motivation among staff to acquire knowledge to provide better care appeared high. We can only surmise as to whether the opportunity for training itself was a key motivator, and such information would be of value in any future study. Implementation of best practices can be difficult to achieve even with multi-pronged approaches, as demonstrated by Irimu and colleagues in their study at a Kenyan tertiary hospital.^17^ Their research pointed to three main enhancing factors for implementation of best practices: ability to mobilise resources, relevance of training to routine work, and emergence of a champion of change. Interestingly, examples of these factors were also found in our study, triggered by the training: (a) District Directorates mobilised resources to establish and operate malnutrition units in two participating hospitals in Ghana, (b) 84% of participants in Ghana and 96% in Latin America reported that the course was of direct relevance to their routine work and (c) champions, primarily nutritionists, emerged in some hospitals enabling effective dialogue with staff and managers. Together these led to policy and operational changes.

Our findings are from health facilities where the introduction of the course was initiated by the local research team. This does not compromise external validity in our view as a similar approach can be used through existing structures within national programmes to improve the management of SAM. The eLearning course can serve as a launch pad, providing knowledge and skills that are accessible at scale without the need for large numbers of trainers. Not all health facility staff took the course and we provided no follow-up practical training, supervision or mentoring. We advise these be included when the course is used in national programmes and we anticipate that their inclusion would lead to further improvements in quality of care and survival. It may be more difficult for isolated learners to change clinical practices, although we know of many determined individuals who have succeeded.

Four of the eleven hospitals already possessed WHO guidelines preintervention but did not follow them fully primarily due to a lack of trained staff, and in many hospitals implementation of the WHO Ten Steps required substantial changes, including provision of equipment and therapeutic feeds, and reorganisation of kitchens. Two hospitals started treating SAM cases postintervention for the first time. Good motivation, cooperation among staff and support from hospital management facilitated the changes made, as reported in other studies.^18^ As has been noted elsewhere, rehydration, charting of feeds, and play activities, were the most difficult practices to change.^19^ In Ghana, negative attitudes of health professionals towards malnourished children changed postintervention, which assisted adoption of improved practices, as noted by others.^20^ In Latin America, preformulated F75 and F100 were not available in the participating hospitals and preparation using WHO recipes was not attempted, possibly because only doctors took the course and they may not have considered such tasks as their responsibility. There were no reports of their sharing knowledge gained from the course with nurses or nutritionists who would normally make feeds.

The study has several limitations, one of which is that Ghana was over-represented among the three countries. Second, the observation period was short and may have failed to capture differences in practices among shifts, weekends etc. Nevertheless, the observed changes are in keeping with the changes reported by the individual health professionals^7^ and senior hospital personnel. Third, we have no precise figure as to the percentage of staff who were trained, as access to the course was open to all and it was used for in-service training in several hospitals after the initial offering of the course as part of the study. A strength of this study is that mortality data cover 12 month periods preintervention and postintervention.

Missed cases increased overall postintervention (5.4% preintervention vs 8.5% postintervention). Many missed cases were from the hospital in El Salvador which considered malnutrition as a complication rather than a diagnosis. El Salvador experienced a severe drought during the postintervention period and the hospital in El Salvador contributed proportionately more to the number of cases assessed against the WHO criteria than it did preintervention. Since this hospital did not record SAM as a diagnosis the number of missed cases increased, and this is reflected by an increase in missed cases for the study as a whole. Excluding this hospital, the proportion of missed cases fell postintervention (4.3% preintervention vs 3.1% postintervention). The proportion of false cases did not improve postintervention. Errors occurred primarily in hospitals using MUAC, including wrongly reporting MUAC=115 mm as SAM.

The main challenge in delivering the course in Latin America is its unavailability in the local language, but this may be temporary as there is interest in providing a Spanish version. In 2018, the eLearning course was re-implemented to enable its access on mobile devices. In Ghana, the anticipated rise in access to mobile devices and improved proficiency in information technology are likely to widen access to the course. Strengths of the eLearning course are that it is convenient, free of cost to the user and can serve as a primer or as a refresher course when needed.

In conclusion, the eLearning course was successful in improving quality of care for children with SAM, leading to a reduction in mortality. We consider the eLearning course to be a scalable tool to train high volumes of in-service and pre-service health professionals with the potential to advance the Sustainable Development Goals.
