# Supplementary figures and images for: Improved care and survival in severe malnutrition through eLearning
Source: Arch Dis Child. 2019 Jul 30;105(1):32–9. doi: 10.1136/archdischild-2018-316539 (PMC6951232; doi:10.1136/archdischild-2018-316539)

## Supplementary file 1

**Figure. Study and data collection periods**

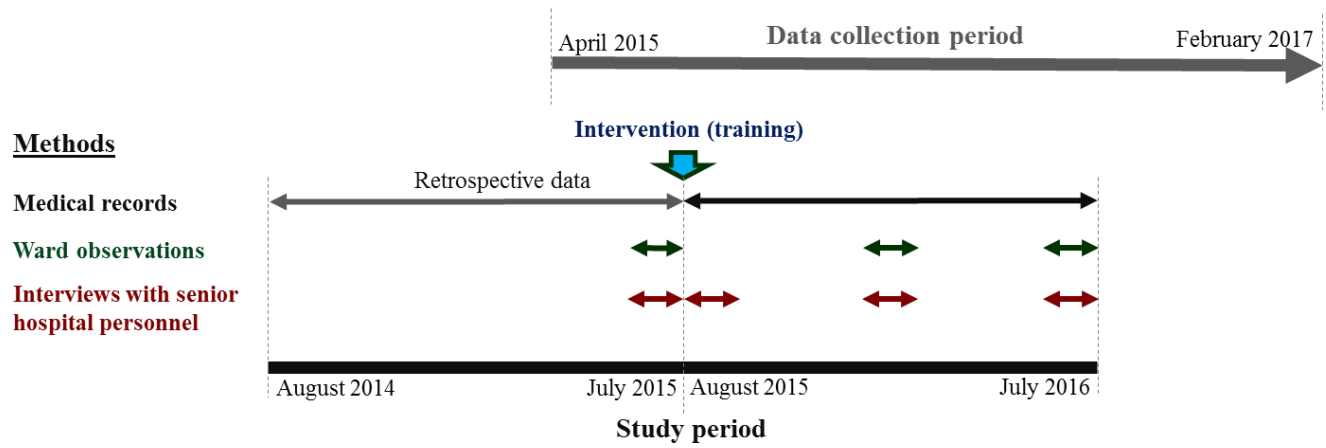

Supplement: Supplementary data [file archdischild-2018-316539supp001.pdf]
