# Supplementary material for: Improved care and survival in severe malnutrition through eLearning
Source: Arch Dis Child. 2019 Jul 30;105(1):32–9. doi: 10.1136/archdischild-2018-316539 (PMC6951232; doi:10.1136/archdischild-2018-316539)
Supplement: Supplementary data [file archdischild-2018-316539supp002.pdf]

Supplementary file 2

Table. Profession, involvement in caring for SAM children, roles and responsibilities in the management of SAM, prior training in SAM management and WHO guidelines preintervention; course use, recommendation of the course to colleagues and retained trained staff postintervention in Ghana

**Hospital staff (H)[1], Total staff trained (T)[2]**	**Hospital[3]**																			
	**MCHH**		**SMiH**		**EGH**		**APH**		**SPH**		**KGH**		**SMaH**		**KSH**		**MGH**		**Total**	
	**H**	**T**	**H**	**T**	**H**	**T**	**H**	**T**	**H**	**T**	**H**	**T**	**H**	**T**	**H**	**T**	**H**	**T**	**H**	**T**
**Overall number of staff trained**	**22**	**31**	**16**	**38**	**6**	**43**	**20**	**37**	**15**	**33**	**13**	**30**	**10**	**35**	**31**	**40**	**8**	**31**	**141**	**318**
**Profession**																				
Doctor/medical officer	1	1	0	0	0	1	0	0	0	0	0	0	0	0	1	1	0	0	2	3
Physician assistant	0	0	0	0	0	0	0	0	0	0	1	1	0	0	1	1	2	2	4	4
Nurses/midwives	14	16	11	20	2	18	10	17	12	14	8	11	7	25	26	34	5	10	95	165
Community health nurses	0	3	0	8	1	8	6	16	0	12	3	7	1	3	1	1	0	9	12	67
Nutritionist	4	5	2	4	2	4	3	3	2	2	1	2	1	3	1	1	0	1	16	25
Public health	2	5	2	2	0	6	1	1	1	2	0	1	1	2	0	0	1	6	8	25
Other	1	1	1	3	1	6	0	0	0	2	0	8	0	2	1	2	0	2	4	26
Not specified	0	0	0	1	0	0	0	0	0	1	0	0	0	0	0	0	0	1	0	3
**Providing care to children with SAM**																				
Yes	20	28	15	34	6	41	16	30	10	24	12	25	10	30	23	31	7	25	119	268
No	2	3	1	3	0	1	1	4	5	7	1	2	0	4	8	9	0	4	18	37
Not specified	-	-	0	1	0	1	3	3	0	2	0	3	0	1	-	-	1	2	4	13
**Roles of staff who were trained[4]**																				
Clinical	17	20	6	10	4	17	9	19	14	18	8	11	2	8	30	35	5	8	95	146
Managerial	4	7	9	14	1	9	2	8	1	4	2	7	4	8	0	2	1	13	24	72
Training	3	3	-	-	0	1	-	-	0	3	1	4	2	4	-	-	0	4	6	19
Policy development	1	1	0	1	-	-	-	-	0	0	-	-	-	-	-	-	0	1	1	3
Monitoring &Evaluation	2	3	0	3	1	8	2	3	0	6	1	3	0	4	0	2	2	13	8	45
Prevention & disease control	0	1	0	5	0	7	3	3	-	-	3	6	5	15	1	1	0	3	12	41
Others, eg. resource allocation	0	3	-	-	-	-	0	1	-	-	0	4	0	2	-	-	0	1	0	11
Not specified	1	1	1	5	0	1	4	4	0	8	0	4	-	1	-	-	0	2	6	26
**Received prior training in SAM**																				
N	12	15	4	6	4	19	7	12	3	10	5	8	4	10	4	8	1	5	44	93
%	54.5	48.4	25.0	15.8	66.7	44.2	35.0	32.4	20.0	30.3	38.5	26.7	40.0	28.6	12.9	20.0	12.5	16.1	31.2	29.2

**Received prior training in the WHO guidelines on the management of SAM**																				
N	7	9	2	2	2	14	3	3	0	1	0	1	1	3	1	2	1	4	17	39
%	31.8	29.0	12.5	5.3	33.3	32.6	15.0	8.1	0.0	3.0	0.0	3.3	10.0	8.6	3.2	5.0	12.5	12.9	12.1	12.3
**Post-intervention**																				
**Overall number of staff responded[5]**	**14**	**15**	**10**	**16**	**-**	**12**	**18**	**29**	**-**	**-**	**7**	**16**	**9**	**16**	**12**	**14**	**6**	**21**	**76**	**138**
**Participants who reported using the course post-intervention**																				
N	10	12	5	12	-	2	14	24	-	-	5	8	7	10	8	10	3	12	52	90
%	71.4	80.0	50.0	75.0	-	16.7	77.8	82.8	-	-	71.4	50.0	77.8	62.5	66.7	71.4	50.0	57.1	67.5	64.7
**Participants who reported to have recommended the course to colleagues**																				
N	7	7	4	11	-	6	12	19	-	-	5	12	8	11	8	10	3	16	47	92
%	50.0	46.7	40.0	68.8	-	50.0	66.7	65.5	-	-	71.4	75.0	88.9	68.8	66.7	71.4	50.0	76.2	61.0	66.2
**Trained staff retained at the participating hospitals post-intervention: confirmed[6]**																				
N	14/22		10/16		-		18/20		-		7/13		9/10		12/31		6/8		76/120	
%	63.6		62.5		-		90.0		-		53.8		90.0		38.7		75.0		63.3	

[1] Limited to the health professionals who specified the hospital name as their work place. Excluded health professionals who stated they worked for Ghana Health Service and nutrition and nursing students on attachments.

[2] Consists of staff working at each participating hopspital, linked community health centres and other health facilities (mostly district level).

[3] MCHH: Maternal and Child Health Hospital, SMiH: St Michael’s Hospital, EGH: Ejura Government Hospital, APH: Agogo Presbyterian Hospital, SPH: St Patrick’s Hospital, KGH: Kogongo Government Hospital, SMaH: St Martin’s Hospital, KSH, Kumasi South Hospital, MGH: Mankranso Government Hospital.

[4] The health professionals were asked to choose options that describe their main responsibilities at work.

[5] The number of health professionals who worked at each participating hospital and consented to participate in the evaluation study of knowledge gained[7]. The actual number of staff trained is greater as the training was open to all.

[6] Estimated number and proportion of the trained staff retained, generated using the numbers of the individual hospital staff who participated in 6m and/or 12m follow-up studies. The data collection of these studies was limited to the staff on duty on the days when the research team visited the hosptials. The actual number of trained staff retained is expected to be higher.

SAM, severe acute malnutrition
