# Supplementary material for: Improved care and survival in severe malnutrition through eLearning
Source: Arch Dis Child. 2019 Jul 30;105(1):32–9. doi: 10.1136/archdischild-2018-316539 (PMC6951232; doi:10.1136/archdischild-2018-316539)
Supplement: Supplementary data [file archdischild-2018-316539supp003.pdf]

Supplementary file 3

Table. Case-fatality rates for SAM for the 12 months preintervention and 12 months postintervention (<6 month, 6-60 month and overall)

Case-fatality rate	Pre-intervention [A]	Post-intervention [B]	Difference [B – A] in % (95% CI)	*P* value
<6m	7/65 (10.8%)	3/83 (3.6%)	-7.2 (-17.3, 1.3)	0.106[3]
6–60m[1]	19/384 (4.9%)	11/662 (1.7%)	-3.3 (-6.0, -1.1)	0.002[2]
Overall	26/449 (5.8%)	14/745 (1.9%)	-3.9 (-6.6, -1.7)	<0.001[2]

[1] Includes matched, unclassifiable and missed SAM.
[2] X^2 test was performed.
[3] Fisher's exact test was performed.
SAM, severe acute malnutrition
