# Supplementary material for: Improved care and survival in severe malnutrition through eLearning
Source: Arch Dis Child. 2019 Jul 30;105(1):32–9. doi: 10.1136/archdischild-2018-316539 (PMC6951232; doi:10.1136/archdischild-2018-316539)
Supplement: Supplementary data [file archdischild-2018-316539supp004.pdf]

Supplementary file 4

Table. Changes in the assessment, diagnosis and management of SAM at eleven participating hospitals for the 12 months preintervention and 12 months postintervention

**Country**		**Ghana**									**Guatemala**	**El Salvador**	**Overall N (%)**
		**Malnutrition ward/units**					**Paediatric & general wards**						
**Healthcare institution[1]**		**MCHH**	**SMiH**	**EGH**	**APH**	**SPH**	**KGH**	**SMaH**	**KSH**	**MGH**	**HJP**	**HNZ**	
**Number of cases analysed: 0-60m**													
Pre	Overall	334	52	41	19	80	206	2503	287	110	133	188	3953
Post	Overall	372	111	55	64	153	238	1782	220	74	212	442	3723
**Number of cases with the requisite measurement data for WHO classification**													
Pre	Overall	165/334	49/52	39/41	14/19	78/80	0/206	0/2503	15/287	0/110	120/133	149/188	629/3953 (15.9%)
Post	Overall	273/372	51/111	49/55	63/64	153/153	33/238	39/1782	0/220	0/74	203/212	436/442	1300/3723 (34.9%)
**Number of cases with reported severe acute malnutrition (SAM)**													
Pre	<6m	38/38	1/6	2/2	2/2	19/19	0/8	0/120	2/17	0/12	1/22	0/46	65/292 (22.3%)
Post	<6m	33/33	16/16	1/4	4/4	27/27	2/6	0/200	0/9	0/3	0/17	0/119	83/438 (18.9%)
Pre	6-60m	293/293	6/46	23/37	12/16	60/60	2/184	0/2383	11/267	0/98	13/111	0/142	420/3637 (11.5%)
Post	6-60m	339/339	93/93	21/51	60/60	126/126	33/229	21/1582	6/211	0/71	20/195	0/323	719/3280 (21.9%)
**Number of cases classified based on WHO SAM criteria (6-60m)**													
**Total number of cases classified[2]**													
Pre	6-60m	293	15	27	12	60	2	0	13	0	17	5	444
Post	6-60m	339	93	23	60	126	33	30	6	0	32	44	786
**Number of unclassifiable SAM**													
Pre	6-60m	138/293	2/15	0/27	2/12	1/60	2/2	0/0	5/13	0/0	1/17	0/5	151/444 (34.0%)
Post	6-60m	72/339	51/93	0/23	1/60	0/126	4/33	0/30	6/6	0/0	1/32	0/44	135/786 (17.2%)
**Number of matched SAM**													
Pre	6-60m	122/293	0/15	20/27	10/12	49/60	0/2	0/0	5/13	0/0	3/17	0/5	209/444 (47.1%)
Post	6-60m	207/339	34/93	16/23	52/60	116/126	13/33	12/30	0/6	0/0	10/32	0/44	460/786 (58.5%)
**Number of false SAM**													
Pre	6-60m	33/293	4/15	3/27	0/12	10/60	0/2	0/0	1/13	0/0	9/17	0/5	60/444 (13.5%)
Post	6-60m	60/339	8/93	5/23	7/60	10/126	16/33	9/30	0/6	0/0	9/32	0/44	124/786 (15.8%)

**Number of missed SAM cases**													
Pre	6-60m	0/293	9/15	4/27	0/12	0/60	0/2	0/0	2/13	0/0	4/17	5/5	24/444 (5.4%)
Post	6-60m	0/339	0/93	2/23	0/60	0/126	0/33	9/30	0/6	0/0	12/32	44/44	67/786 (8.5%)
**Mortality**													
**Number of deaths from all causes (number of deaths/number of cases analysed 0-60m)**													
Pre	Overall	N/A	N/A	N/A	N/A	N/A	3/206	0/2503	4/287	0/110	0/133	0/188	7/3427 (0.2%)
Post	Overall	N/A	N/A	N/A	N/A	N/A	0/238	1/1782	0/220	0/74	0/212	0/442	1/2968 (0.03%)
**Case fatality rates for children with SAM**[3]													
Pre	Overall	12/298	0/12	4/26	0/14	6/69	0/2	0/0	4/14	0/0	0/9	0/5	26/449 (5.8%)
Post	Overall	2/312	1/101	0/19	3/57	8/143	0/19	0/21	0/6	0/0	0/23	0/44	14/745 (1.9%)
**SAM-related morbidity**													
**Number of cases diagnosed with acute respiratory infection in whom SAM was identified**													
Pre	Overall	38/44	0/8	1/1	0/0	0/0	1/29	0/377	1/52	0/39	0/3	3/67	44/620 (7.1%)
Post	Overall	23/23	17/18	0/0	0/0	16/17	3/41	0/314	1/81	0/22	1/27	20/199	81/742 (10.9%)
**Number of cases diagnosed with gastroenteritis in whom SAM was identified**													
Pre	Overall	22/25	1/5	2/2	1/1	3/3	0/18	0/342	1/34	0/22	1/10	3/125	34/587 (5.8%)
Post	Overall	13/13	20/22	0/0	0/0	32/34	0/14	1/351	0/57	0/15	5/43	24/167	95/716 (13.3%)
**Other outcome change**													
**Number of SAM admissions referred from Paediatric/Malnutrition wards to Komfo Anokye Teaching Hospital (Ghana only)**													
Pre	Overall	28/298	0/12	0/26	0/14	0/69	0/2	0/0	1/14	0/0	N/A	N/A	29/435 (6.7%)
Post	Overall	8/312	0/101	0/19	0/57	1/143	0/19	0/21	0/6	0/0	N/A	N/A	9/678 (1.3%)

[1] MCHH: Maternal and Child Health Hospital, SMiH: St Michael's Hospital, EGH: Ejura Government Hospital, APH: Agogo Presbyterian Hospital, SPH: St Patrick's Hospital, KGH: Kogongo Government Hospital, SMaH: St Martin's Hospital, KSH, Kumasi South Hospital, MGH: Mankranso Government Hospital.

[2] Include reported SAM cases and cases with no SAM reported but of which anthropometric data met WHO SAM criteria.

[3] Include SAM cases aged 6-60m that met WHO criteria (matched, unclassifiable, missed), and SAM cases aged <6m.

SAM, severe acute malnutrition
