# Supplementary material for: Improved care and survival in severe malnutrition through eLearning
Source: Arch Dis Child. 2019 Jul 30;105(1):32–9. doi: 10.1136/archdischild-2018-316539 (PMC6951232; doi:10.1136/archdischild-2018-316539)
Supplement: Supplementary data [file archdischild-2018-316539supp005.pdf]

Supplementary file 5

Excerpts. Taken from an interview with the Director of the Ahafo Ano South District Health Directorate, illustrating some of the changes in a district with 1 hospital and 11 health centres.

In relation to screening: '*...Previously they(staff) were not using the MUAC… they were just using the underweight, comparing their weight to their age to refer cases but after the training, as I said before, there's been substantial increase in the number of cases that are identified and referred. So, the practices have changed in our corners with the new knowledge acquired from the training…'*

In relation to malnutrition prevalence: '… *what we've been doing is that we have a community nutrition surveillance system. Compared to the previous year (pre-training), there was improvement in terms of malnutrition in the communities.*

In relation to improved outcomes: '*... for now rehabilitation rate has increased from about 80% to about 95%. And with the mortality rate, even though it is insignificant in terms of numbers, I think it has decreased to about 50% of what we used to see before the training…'*

And in relation to improved staff competence: '*... (At community level) ...I can say confidently that some of them (staff) are applying the knowledge they acquired from the training in terms of managing cases…they (staff) are more competent now, I've seen a lot of improvement in that the cases are being responsive to treatment. And now I think they are using the (WHO) steps very well.'*
